# Supplementary material for: CXCL13/CXCR5 Interaction Facilitates VCAM-1-Dependent Migration in Human Osteosarcoma
Source: Int J Mol Sci. 2020 Aug 24;21(17):6095. doi: 10.3390/ijms21176095 (PMC7504668; doi:10.3390/ijms21176095)
Supplement: Supplementary file 1 [file ijms-21-06095-s001.pdf]

**Supplementary Materials**

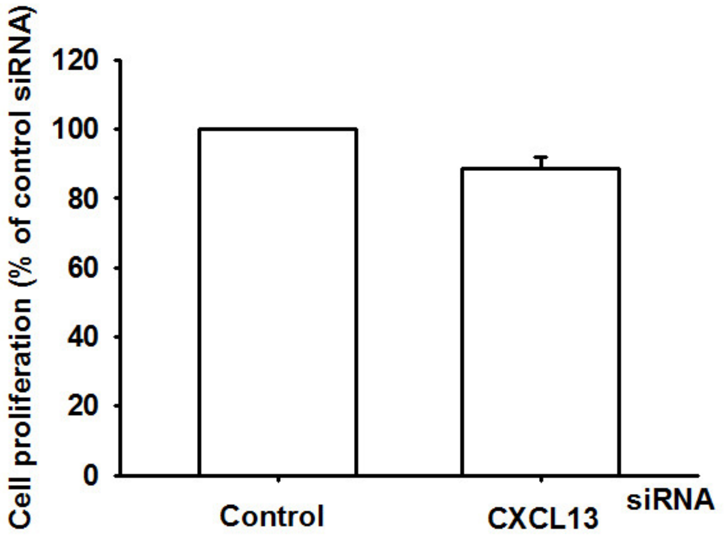


**Figure S1.** Knockdown of CXCL13 did not affect osteosarcoma cell proliferation. No significant difference was seen in levels of proliferation between MG-63 cells transfected with control siRNA and MG-63 cells transfected with CXCL13 siRNA. Data represent the mean ± S.D.

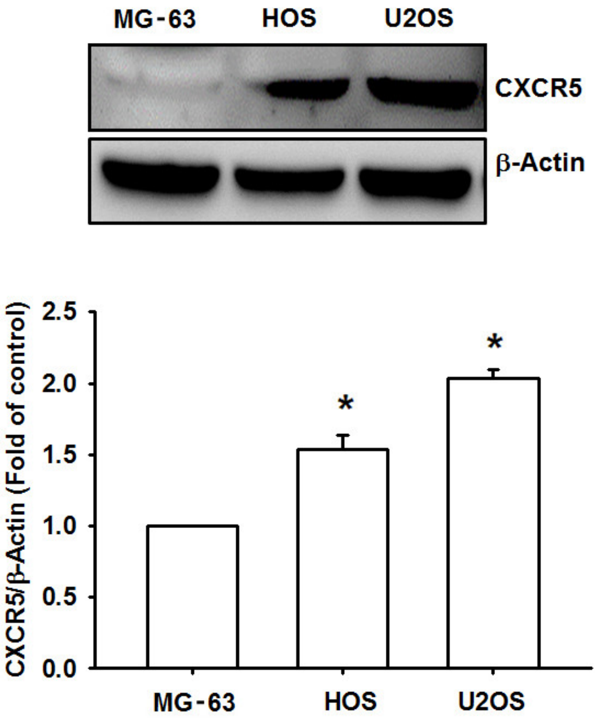


**Figure S2.** Levels of CXCR5 protein expression in three osteosarcoma cell lines (MG-63, HOS,

U2OS). The findings suggest that CXCR5 expression correlates with malignancy. Clearly, CXCR5 expression was very high in the U2OS and HOS cells. *$p < 0.05$ compared with the MG-63 cells.

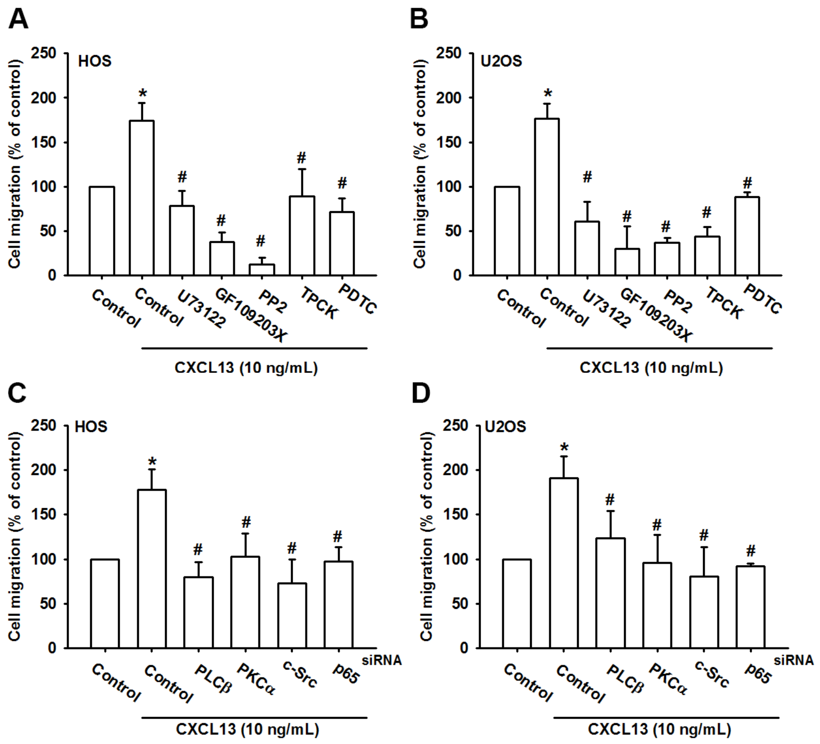


**Figure S3**. The PLCβ, PKCα, c-Src and NF-κB signaling pathways facilitate increases in osteosarcoma cell migration induced by CXCL13. (**A**–**D**) HOS and U2OS cells were pretreated with U73122, GF109203X, PP2, PDTC, and TPCK, or transfected with PLCβ, PKCα, c-Src and p65 siRNAs, then stimulated with CXCL13; migratory potential was examined by the Transwell assay. *$p < 0.05$ compared with the control group; #$p < 0.05$ compared with the CXCL13-treated group.

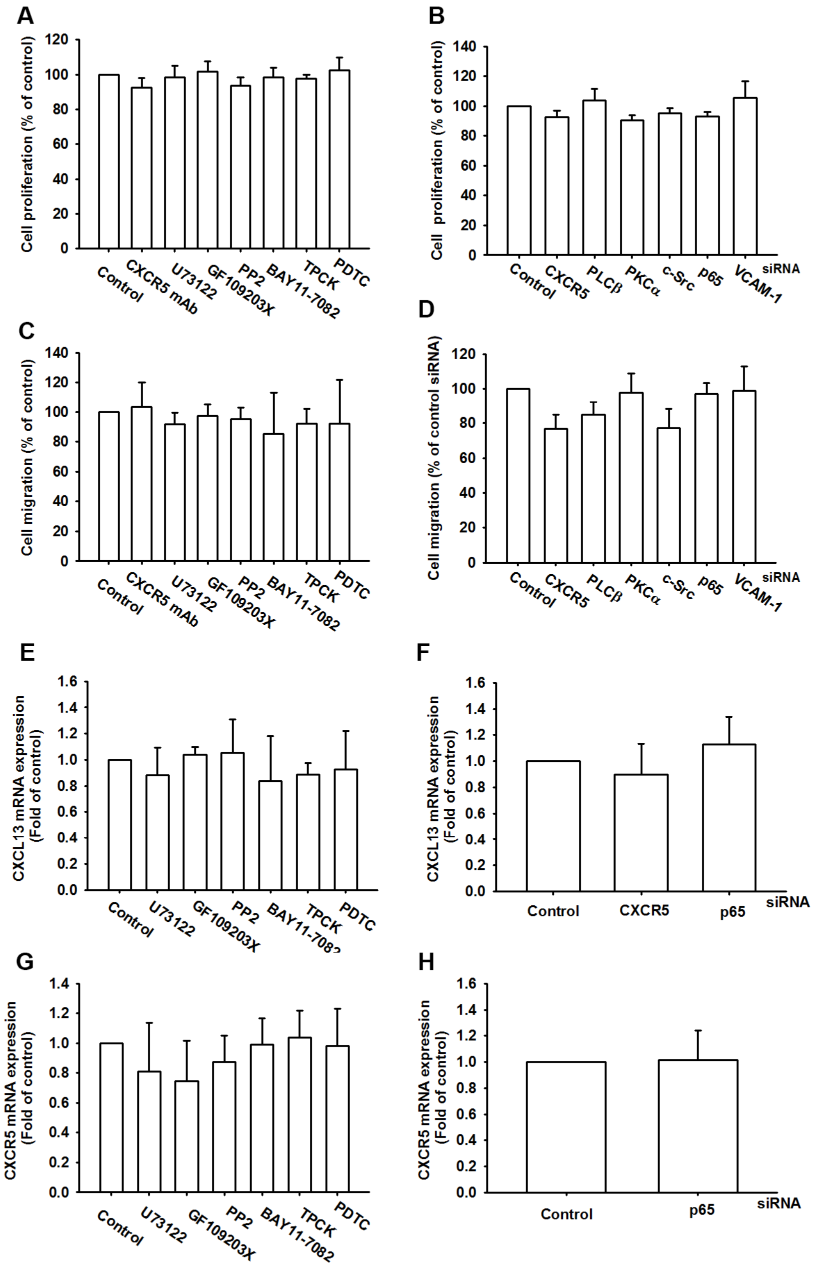


**Figure S4.** Impacts upon MG-63 cell proliferation and migration after treatment with PLCβ, PKC$\alpha$, c-Src and NF-κB inhibitors or transfection with their respective siRNAs. (A,B) The MTT assay results revealed no significant impacts upon cell proliferation, which suggests that these treatments did not affect cell viability. (C,D) Cell migration, (E,F) CXCL13 expression, (G,H) CXCR5 expression was not affected by any of the above treatments or transfection with their respective siRNAs. Data represent the mean ± S.D.
